# Hsp90 Inhibitors and the Reduction of Anti-Cancer Drug Resistance by Non-Genetic and Genetic Mechanisms

**DOI:** 10.3390/ph5090890

**Published:** 2012-08-30

**Authors:** Xiangyi Lu, Luan Wang, Douglas M. Ruden

**Affiliations:** 1 Institute of Environmental Health Sciences, Wayne State University, Detroit, MI 48201, USA; Email: xlu@wayne.edu (X.L.); du1484@wayne.edu (L.W.); 2 C. S. Mott Center for Human Growth and Development, Department of Obstetrics and Gynecology, Wayne State University, Detroit, MI 48201, USA

**Keywords:** Hsp90, genetic capacitor, nuclear hormone receptors, cancer, aneuploidy, epigenetics, DNA methylation, chaperones, transcriptional pausing

## Abstract

In this review, we focus on how inhibitors of Hsp90 can help prevent the resistance to anti-cancer drugs by synergistically increasing their cancer killing abilities and thereby allowing them to function at much lower concentrations than normally used. Hsp90 helps to fold numerous client proteins, such as Akt, Raf, Src, chromatin-modifying proteins, nuclear hormone receptors, and kinetochore assembly proteins. We discuss four mechanisms by which Hsp90 inhibitors can potentially synergize with anti-cancer drugs: by making a drug-resistant protein that is a client for Hsp90 more sensitive to the drug, by increasing chromosomal aneuploidy and the effectiveness of DNA-damaging drugs, by inhibiting Trithorax proteins which trimethylate histone 3 at lysine 4 (H3K4me3) and thereby decreasing expression of tumor promoter genes, and by interacting with the negative elongation factor (NELF) complex in tumors. We also explain how the evolutionary capacitor function of Hsp90 can be exploited with inhibitors of Hsp90 by exposing new protein variants that can be targeted with other drugs, thereby opening new avenues of combination drug therapy to treat cancer. We believe that inhibition of these processes can increase the efficacy of Hsp90 inhibitors with other anti-cancer drugs.

## 1. Introduction

The acquisition of drug resistance in cancer cells might at first appear to be a Lamarckian process because drug-resistance is a characteristic that was not present in the primary cancer cells. However, most mechanisms of drug resistance focus on genetic mutations or amplifications in the genes encoding the proteins that are targeted by the drug. Such mutations or amplifications are “normal” Mendelian processes in the sense that the drug-resistance phenotype involves the inheritance of mutant alleles. In this review we attempt to focus on Lamarckian-sounding ideas for acquiring drug resistance by non-genetic means by explaining how Hsp90 inhibitors might synergize with other anti-cancer drugs at the transcriptional or protein level by impeding its chaperone activities and by releasing its capacitor functions.

First, we will briefly discuss a model from the father of evolutionary theory, Charles Darwin, for how an organism might inherit acquired characteristics. In his book *The Variation of Animals and Plants under Domestication* [1], which was published nine years after his *On the Origin of Species* [2]. Darwin proposed that “gemmules” were secreted by the organs of the body after adapting to a stressful environment and, via transport through the blood, accumulated in the germ cells. Through these gemmules, Darwin proposed a mechanism to explain Lamarck’s 1809 theory for the inheritance of acquired characteristics [3]. Interestingly, research in the past couple of decades has shown that Darwin’s notional gemmules have many interesting similarities to nuclear hormones, some of which, such as glucocorticoids, are induced by stress, travel via the blood, and influence somatic properties such as development and cancer [4]. Furthermore, endocrine disruptors such as diethylstibestrol (DES), which is more potent than the hormone estrogen in binding to and activating the estrogen receptor, have been shown to increase the incidence of cervical cancer in women for several generations (reviewed in [5]). This is relevant to the topic of this paper because drugs that target the estrogen receptor and other nuclear hormone receptors, such as tamoxifen, are some of the mostly commonly-used drugs to treat breast cancer.

The predominant idea in the cancer field is that Hsp90 inhibitors synergistically help kill cancer cells treated with drugs that inhibit Hsp90 client proteins because inhibition of Hsp90 generally reduces the levels of the client proteins. However, the focus of this review is on the role of Hsp90 in facilitating not only genetic but also non-genetic changes in the genome during stress, such as occurs during cancer progression, and how inhibitors of Hsp90 can synergize with other anti-cancer drugs. Hsp90 is a chaperone protein that is required for the proper folding of over 200 signaling molecules, including many oncogenes, such as Raf and Src, and chromatin-regulatory genes, such as Trithorax and Smyd3, which induce the H3K4me3 histone mark that is associated with actively transcribed genes [6,7,8]. Hsp90 functions as a capacitor for phenotypic variation via both genetic [9,10] and non-genetic means [11,12]. A capacitor, in the biological sense, is something that holds back cryptic (*i.e*., hidden) phenotypic variation which becomes manifest in stressful environments [13]. Genetic capacitors have also been called “adaptively inducible canalizers” by Meikeljohn and Hartl [14] and “Waddington’s widget” in a review from our laboratory [11]. We believe that it is the disruption of the capacitor function of Hsp90 that allows Hsp90 inhibitors to potentiate the activity of other anti-cancer drugs. In the next section, we review what is known about the capacitor function of Hsp90.

## 2. Hsp90 as a Capacitor for Phenotypic Variation and Cancer Cell Survival

Hsp90 is an evolutionarily conserved protein that forms a ring-like dimer when it binds and facilitates the proper folding of its client proteins. Hsp90 has been called a “master regulator of master regulators” [15] and the “hub of protein homeostasis” [6] because of its key function in regulating numerous signaling molecules. Rutherford and Lindquist, in their classic paper “Hsp90 as a Capacitor for Morphological Evolution” [13], proposed that Hsp90 is a key component of a system that allows organisms to adapt to stressful environments. They showed that *Drosophila melanogaster* laboratory strains that have reduced Hsp90 function due to heterozygosity for several different mutations in the *Hsp83* gene displayed a variety of morphological abnormalities at low frequencies in a least several different body parts [13]. After several generations of selection for these abnormal phenotypes, eventually nearly 100% of the flies had the morphologically abnormal phenotype, even when Hsp90 activity was fully restored by adding back the wild-type *Hsp83* gene.

Similar results were obtained when Hsp90 function was inhibited by its potent and specific drug inhibitor geldanamycin, derivatives of which are currently used in several cancer studies [13]. Lindquist and colleagues called Hsp90 a “capacitor” for morphological variation because inhibition of Hsp90 activity resulted in depletion of developmental buffering, akin to the electrical charge depletion by an electrical capacitor, so that a variety of morphological abnormalities would appear in the offspring [13].

What Rutherford and Lindquist [13] did in 1998, as recognized by McLaren [16] and our laboratory [11] in later reviews, essentially was to repeat and update classical experiments done by Waddington some 50 years earlier. Charles Waddington is often called “the father of epigenetics” partly because he popularized the word epigenetics, at least as it applies to development. He showed that stressing *D. melanogaster* larvae, such as by heat shock, can cause a small percentage of adults to emerge that have a novel phenotype, such as missing crossveins in the wing (*i.e*., the crossveinless phenotype) [17]. Waddington defined epigenetics as “the interaction of the genome with the environment” [18], but more recent definitions of epigenetics specify cellular biochemical mechanisms such as histone modifications, small RNAs, and even prions (reviewed in [19]).

As with Rutherford and Lindquist’s experiments [13], Waddington observed that after more than 20 generations of heat-shock and re-selection, eventually 100% of the offspring had the crossveinless or Ultrabithorax phenotype, depending on which phenotype had initially been selected. As mentioned above, the novel phenotypes were stabilized so that environmental stress was no longer necessary for the offspring to have these phenotypes.

In modern molecular terms, what is likely happening in Waddington’s classic experiments is that several signaling molecules become partially inactivated by stress. In a population, allelic variation would ensure that some variants of signaling molecules would be more susceptible to stress than other signaling molecules (e.g., they denature at a lower temperature). According to Lindquist and colleagues, in stressful conditions, the Hsp90 pool could become titrated by binding to the partially denatured proteins [13]. The remaining Hsp90 proteins would be insufficient in number relative to its many native-folded client-signaling proteins, with the result that they would become inactivated due to the lack of available Hsp90 protein [13].

In normal cells, over 90% of Hsp90 is in an inactive monomer form. However, in cancer cells, a similar proportion of Hsp90 dimers are bound to client proteins, such as over expressed onco-proteins. Such cancer cells are often described as being “oncogene addicted” because inactivation of the oncogenes would quickly lead to cell death via the induction of apoptosis [20]. One interesting attribute of Hsp90 dimers bound to client proteins is that this form of Hsp90 is one or more orders of magnitude more sensitive to Hsp90 inhibitors such as geldanamycin than the monomeric form of Hsp90 [20]. Therefore, even though all cells contain as much as 1–2% Hsp90 protein, cancer cells are nevertheless selectively killed by Hsp90 inhibitors compared with normal cells [20]. Such selectivity of Hsp90 to cancer cells, which presumably does not occur with most other anti-cancer drugs, might help explain how Hsp90 inhibitors synergistically function with other anti-cancer drugs. Therefore, treating patients with Hsp90 inhibitors would decrease the levels of many proteins that are targets of other anti-cancer drugs, such as Raf, Src, and the EGR Receptor. Furthermore, through its capacitor function, Hsp90 inhibitors would reveal previously cryptic genetic information and thereby generate new protein targets for combination anti-cancer drug therapy.

## 3. Hsp90 and the Generation of Chromosomal Aneuploidies

Recently, Chen and colleagues showed that Hsp90 inactivation by stress potentiates rapid cellular adaptation through induction of aneuploidy and drug resistance [21]. Aneuploidy is defined as a departure in chromosomal number from normal diploidy (in somatic cells) or from normal haploidy (in germ cells). Aneuploidy may be observed on an evolutionary timescale—especially in plants, where whole genome duplications and selective chromosome loss commonly distinguish closely related species. Aneuploidy is also a hallmark of cancer. Recent studies have shown that aneuploidies are a form of genome-scale changes that can confer adaptive phenotypes under diverse stressful environments.

Chen and colleagues showed that the Hsp90 inhibitor geldanamycin, among dozens of drugs tested, is the best inducer of aneuploidy in the budding yeast *S. cerevisiae* [21]. The chaperone function of Hsp90 has an evolutionarily conserved role in kinetochore assembly. The kinetochore is the centromeric region of the chromosome that attaches to the microtubule spindle during mitosis. Reduced Hsp90 activity thus destabilizes the chromosomal attachment to the mitotic spindle, which would facilitate non-disjunction or metaphase chromosome loss. Chen and colleagues used a heterozygous genetic mutation on chromosome XV that causes sensitivity to a drug, which can be suppressed by adding an additional copy of the wild type gene via duplication the non-mutated chromosome XV. These findings demonstrate that aneuploidy is another type of stress-induced mutation and reveals a new role for Hsp90 as a capacitor for genetic variation.

Many cancers have mutations in DNA repair enzymes as driver genes, such as 0^6^-methylguanine methyltransferase (MGMT) mutations in the gene that repairs 0^6^-methylguanine modified DNA [22]. While Chen and colleagues showed that Hsp90 inhibitors can increase drug resistance in yeast, it is also possible that Hsp90 inhibitors reduce drug resistance in cancer cells to DNA damaging agents such as cisplatin. The genome instability induced by Hsp90 inhibitors in cancer cells could, for instance, make the cancer cells hypersensitive to anti-cancer drugs that damage the DNA [23,24].

Additionally, DNA damaging drugs might in turn increase the effectiveness of Hsp90 inhibitors. One well-documented response of treating cells, either normal or cancer cells, with Hsp90 inhibitors is that the stress response (e.g., an increase in Hsp70 protein levels) is induced in these cells [25,26]. This is an unfortunate side-effect of Hsp90 inhibitors because the stress response allows cells to survive in stressful environments, such as in the presence of other anti-cancer drugs. However, cisplatin has been shown to abrogate the stress-response in Hsp90 inhibitor treated cells [27]. Therefore, Hsp90 not only increases the efficacy of some other anti-cancer drugs, but other anti-cancer drugs also increase the efficacy of Hsp90 by diminishing the stress response.

## 4. Hsp90 and the Initiation of Polymerase Pausing

In a recent review, we described how several laboratories have shown that Hsp90 inhibitors in combination with other anti-cancer drugs can allow much lower doses of the other drugs to be used to kill cancer cells [15]. In a paper that came out after our review, Paraiso and colleagues showed that the Hsp90 inhibitor XL888 overcomes resistance to the BRAF inhibitor vemurafenib [28]. Similarly, Weigert and colleagues showed that Hsp90 inhibitors significantly enhance the efficacy of JAK2 inhbitors [29] (reviewed in [30]). Also, Best and colleagues showed that the Hsp90 inhibitor SNX-7081 synergizes with and restores sensitivity to fludarabine in chronic lymphocytic leukemia that are mutated for TP53 (Human P53) [31].

**Figure 1:**
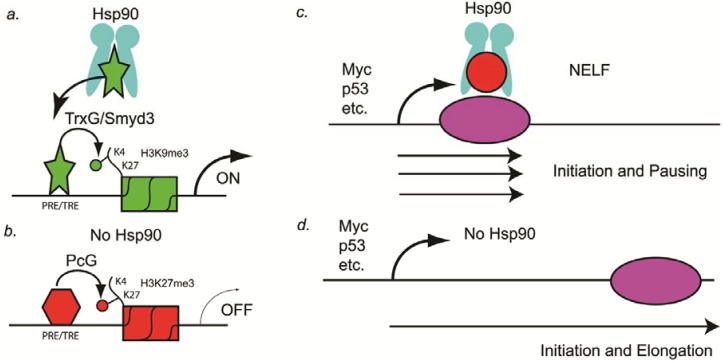
Hsp90 as a genetic and epigenetic capacitor for phenotypic variation. (**A**) In a non-stressed cell, Hsp90 binds to the H3K4 histone methyltransferase (Trx or Smyd3) and activates the enzymatic activity. Chromatin of PRE/TREs carrying this active histone mark is open for transcriptional activation; (**B**) In a stressed cell, Hsp90 is inactive and Trx is not activated. Consequently, PRE/TREs are occupied by PcG complex proteins, one of which is a H3K27 histone methyltransferase (PcG). Chromatin thus modified by this inactive histone mark leads to transcriptional repression; (**C**) In the presence of Hsp90, transcription pauses at as many as 30% of the genes, including the oncogene Myc and the tumor suppressor gene p53. Pausing is mediated by NELF (red circle), which is a target of Hsp90; (**D**) In the absence of Hsp90, NELF no longer induces pausing and transcriptional elongation occurs. Elongation of tumor suppressor genes such as p53 in cancer cells can lead to stasis or apoptosis, especially in combination with other anti-cancer drugs.

In recent research on the role of Hsp90 during *Drosophila* development, the Paro laboratory demonstrated two possible mechanisms that might explain how Hsp90 inhibitors might synergize with anti-cancer drugs and thereby overcome resistance to those drugs. The first mechanism they showed is that Hsp90 binds to Trithorax protein in the promoters of many genes during *Drosophila* development (Figure 1A) [32]. Trithorax functions as an epigenetic memory system by inducing trimethylation of histone 3 lysine 4 (H3K4me3), which is present in genes that are undergoing transcription [32]. Therefore, in the absence of Hsp90, a general reduction in transcription should occur (Figure 1B). This general reduction in transcriptional activation of genes such as oncogenes could be one mechanism by which Hsp90 inhibitors synergize with anti-cancer drugs.

The second mechanism discovered in the Paro laboratory that might explain how Hsp90 inhibitors might function in synergizing with other anti-cancer drugs is by regulating pausing of RNA polymerase II. Sawarkar and colleagues in the Paro laboratory showed that Hsp90 is bound to negative elongation factor (NELF) complex at the promoters of paused genes and causes abortion of transcription (Figure 1C) [33]. Originally, paused genes were thought to be rare, such as at heat shock genes, but recently Nechaev and Adelman showed that as many as 30% of human genes have paused transcripts [34]. In *Drosophila*, several of the paused genes identified by the Paro laboratory include oncogenes and tumor suppressor genes such as c-myc, P53, Notch, Delta, Serrate, TGFalpha (vein, spitz, and gurken), wingless, and several others [33]. Therefore, in the absence of Hsp90, tumor suppressor genes such as P53 could be activated and thereby lead to cancer cell stasis or the induction of apoptosis (Figure 1C).

## 5. Conclusions

In this review, we discussed the role of Hsp90 and stress in regulating evolution by genetic and non-genetic mechanisms. As discussed earlier, many evolutionary biologists have disputed the importance of Hsp90 as a capacitor for morphological evolution. However, the original paper of Rutherford and Lindquist in 1998 [13] has stood up very well, and the role of Hsp90 as an adaptively inducible canalizer is gaining more and more prominence among evolutionary and cancer biologists [35]. We believe that studies of Hsp90’s capacitor function, such as by preventing aneuploidy and by interacting with transcription complexes, will continue to penetrate into the cancer biology community, and will eventually be seen as a cornerstone for understanding gene-environment interactions.
